# In Situ Thermography of the Metal Bridge Structures Fabricated for the 2018 Additive Manufacturing Benchmark Test Series (AM-Bench 2018)

**DOI:** 10.6028/jres.125.005

**Published:** 2020-01-30

**Authors:** Jarred C. Heigel, Brandon Lane, Lyle Levine, Thien Phan, Justin Whiting

**Affiliations:** 1 National Institute of Standards and Technology,Gaithersburg, MD 20899, USA

**Keywords:** 3D build, additive manufacturing, benchmark tests, Additive Manufacturing Benchmark Test Series, AM-Bench 2018, IN625, nickel superalloy 625, powder bed fusion, temperature measurement, thermography

## 1 Summary

This document provides details on the files available for download in the data set "In situ thermography of the metal bridge structures fabricated for the 2018 Additive Manufacturing Benchmark Test Series (AM-Bench 2018)". The experiments were performed to support the 2018 AM-Bench^1^1
https://www.nist.gov/ambench Class 01 experiments consisting of metal three-dimensional (3D) builds. The modeling community was invited to predict the following: (1) part deflection, (2) residual elastic strains, (3) microstructure, (4) phase fractions, and (5) phase evolution. Details for these proposed challenges and the postprocess measurement results can be found at their respective links on the AM-Bench website.^1^

This document describes the experiments conducted on a commercial laser powder bed fusion (LPBF) machine and the *in situ* thermography and explains the resulting measurement data. The purpose of disseminating these data is twofold: (1) to provide layer-wise thermal history data for part-scale model validation and (2) to provide insight into the thermal history responsible for the postprocess distortion, residual strain, and microstructure. Measurements are reported in radiance temperature. The calculation of true temperature for direct comparison with model results requires knowledge of the effective surface emissivity. At this time, the effective emissivity is unknown, but assumptions can be applied based on the literature. The following sections detail the experiment and measurement setup, describe the data files, and provide equations to enable the calculation of true temperature from the measured radiant temperature.

## 2. Data Specifications

**Table:** 

**NIST Operating Unit(s)**	Engineering Laboratory
**Format**	There are several types of data formats included in this data set. Please refer to Sec. 4 for a description of each type of data.
**Instruments**	An EOSint M270D^a^ laser powder bed fusion system was used to fabricate the bridge structures. An IRCamera model IRC 912 infrared camera was used to perform thermography of the scan tracks. Details are provided in Sec. 3.
**Spatial or Temporal Elements**	These measurements were performed on January 31-February 2, 2018
**Data Dictionary**	N/A
**Accessibility**	All data sets^b^ submitted to *Journal of Research of NIST* are publicly available.
**License**	https://www.nist.gov/director/licensing

a Certain commercial equipment, instruments, or materials are identified in this paper in order to specify the experimental procedure adequately. Such identification does not imply recommendation or endorsement by the National Institute of Standards and Technology (NIST), nor does it imply that the materials or equipment identified are necessarily the best available for the purpose.

b NIST uses its best efforts to deliver a high-quality copy of the database and to verify that the data contained therein have been selected on the basis of sound scientific judgment. However, NIST makes no warranties to that effect, and NIST shall not be liable for any damage that may result from errors or omissions in the database.

## 3 Experiment Method

**Fig. 1:**
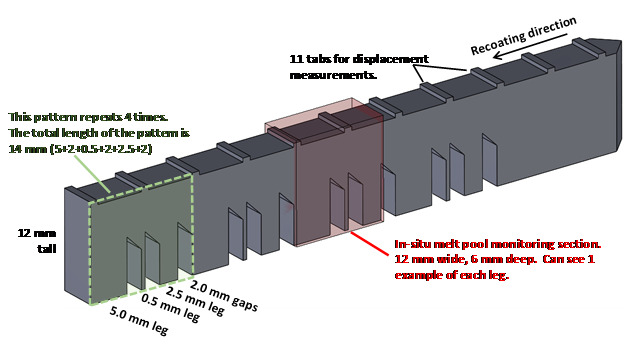
The AMB2018-01 bridge structure geometry.

The experiment consists of using a commercial LPBF system to manufacture metal alloy (nickel superalloy 625 and stainless-steel 15-5) bridge structures. The bottom half of the structure consists of 12 legs of varying size and a larger base. The top half of the structure consists of a single bridge section that connects the legs to the base, as shown in Fig. 1. A high-speed infrared (IR) camera is used to measure the thermal history of each layer within a small region of interest (ROI) of the part that contains one example of each of the three different leg sizes. This strategy enables the thermal history of each of the leg sizes and bridge section to be measured so that the result can be correlated with the postprocess measurements of microstructure, strain, and distortion.

While the primary objective of the experiments was to produce parts for the postprocess analysis of distortion, strain, and microstructure, the purpose of the *in situ* measurements of thermal history was to provide greater insight into the phenomena observed in the postprocess measurements listed above and to provide thermal data for model validation. The following sections explain the *in situ* thermography setup (Sec. 3.1), the build geometry (Sec. 3.2), the materials (Sec. 3.3), and the build strategy (Sec. 3.4).

## 3.1 *In Situ* Infrared Temperature Measurement Description

**Fig. 2:**
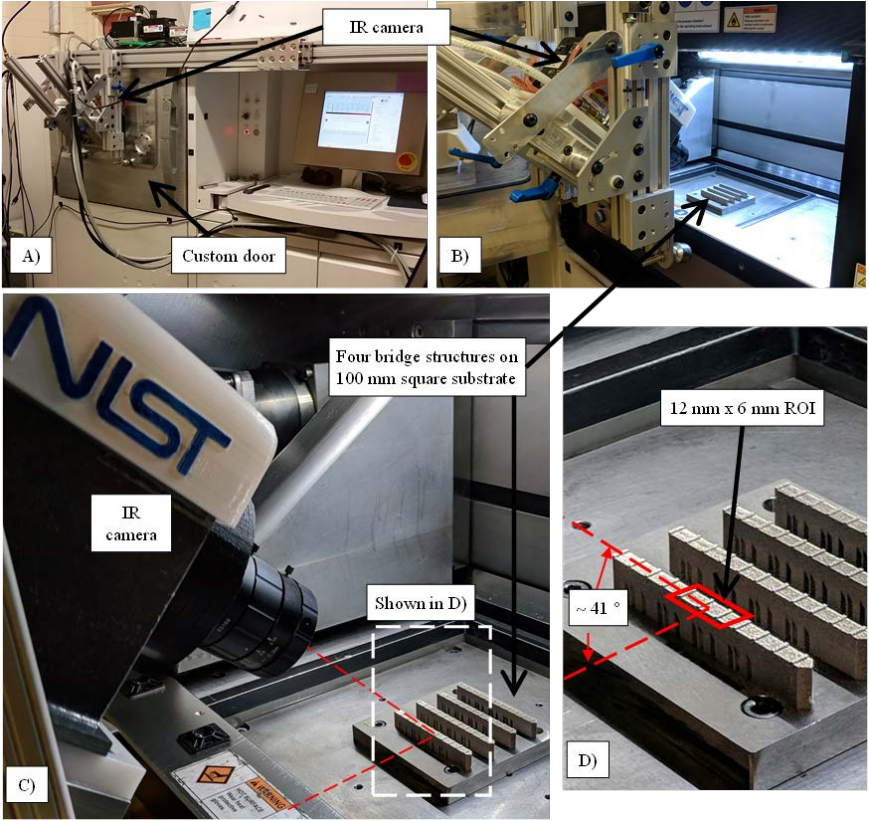
Images depicting the experiment setup. (A) The EOSint M270D power bed fusion system with the custom door and IR camera. (B-C) The system with the door open, revealing the relative positioning of the IR camera to the 100 mm square substrate with four bridge structures. (D) A magnified view of (C) showing the ROI observed by the camera.

Figure 2 presents the experiment setup used in this study. A custom door was fabricated and mounted to the EOSint M270D, as originally presented by Lane *et al*. [1]. The custom door enabled the IR camera to be positioned as close to the build as possible to allow higher magnification. The camera was mounted to an articulating frame attached to the machine. When positioned, the camera was approximately 162 mm from the ROI and was angled approximately 41° from the build plane.

The IR camera implemented in this study was an IRCamera model IRC 912. A band-pass filter was installed in the built-in filter wheel to limit the detectable wavelength range from 1350 nm to 1600 nm. Filtering served two purposes: (1) The light equal to the laser wavelength of 1070 nm must be blocked from the camera, and (2) a narrow range of wavelengths minimizes possible errors that may arise from an incorrect assumption of a wavelength-independent emissivity value (gray-body assumption). The integration time (shutter speed) of the camera was 40 µs, and the frame rate was 1800 frames per second. This frame rate is the maximum possible for the camera before significant numbers of frames are dropped, and the chosen integration dictates the measurable radiant temperature range. To achieve this frame rate, a limited window size was used (360 horizontal pixels, 126 vertical pixels). Considering the camera magnification of approximately 0.33×, the working distance of approximately 162 mm, and the relative angle between the camera and the target surface of 41°, the instantaneous field of view (iFOV, or pixel resolution) in the horizontal and vertical axes were approximately 34 µm and 52 µm, respectively. Please note that these values may have changed slightly for each build, since the camera was repositioned between builds to allow access to the build chamber. The actual iFOV of each build is provided in the data files.

The measured camera signal is related to the temperature of the object according to [2]:

Smeas=ε FTbb=F(Trad) (1)

where $S_{\text{meas}}$ is the camera signal in digital levels (DLs), $T_{\text{bb}}$ is temperature in K, and ε is the effective emissivity of the object. Effective emissivity is a dimensionless value between 0 and 1. Only for perfectly emitting black bodies does ε= 1; all other bodies emit a fraction of the radiation. Consequently, the camera measures a signal in response to this radiated temperature, $T_{\text{rad}}$ in K, and the true temperature of the object can be calculated only if ε is known. The function relating $T_{\text{rad}}$ to $S_{\text{meas}}$ is defined by the Sakuma-Hatori equation and its inverse [3]:

$F\left(T_{\text{rad}}\right)=S_{\text{meas}}=\frac{C}{\exp\left(\frac{c_{2}}{AT_{\text{rad}}+B}\right)-1}$ (2)

and

**Fig. 3:**
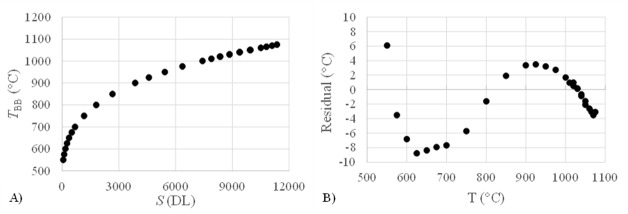
Results of the black body calibration. (A) The relationship between average camera signal and black-body temperature. (B) The residual from fitting Eq. (1) to the data presented in (A).

F-1S=Trad=c2AlnCS+1-BA (3)

where ${\text{c}}_{2}$ is the second radiation constant (14 388 μm/K), and the coefficients *A*, *B*, and *C* are determined via the black-body calibration procedure outlined by Lane and Whitenton [2]. A black body is first used to create a two-point nonuniformity correction (NUC), and then series of measurements are performed using the black body incrementally set to a range of temperatures covering the detectable range of the camera (550 °C to nearly 1100 °C), which is a function of the camera settings and optical system. Figure 3 presents the results of this calibration, where the black-body temperature, $T_{\text{bb}}$, is plotted against the average camera signal over 100 frames. The coefficients *A* = 2.665, *B* = -800.7, and *C* = 1.94x10^6^ are determined by assuming ε= 1 and fitting Eq. (2) to the data presented in Fig. 3A. The residuals of this fit are presented in Fig. 3B, while the root-mean-square error (RMSE) of the fit is 8.1 °C. The RMSE is an estimate of the calibration uncertainty.

## 3.2 Part Design

**Fig. 4:**
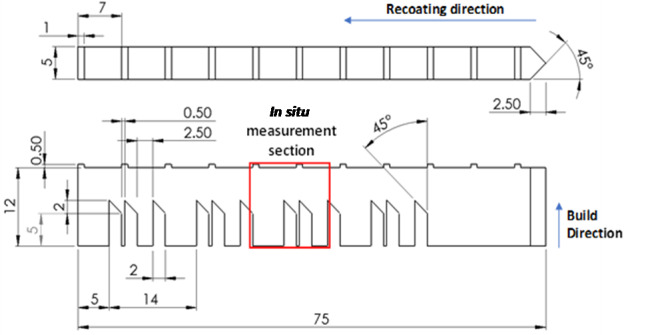
Plane (top) and elevation (bottom) views of the bridge structure geometry. Linear dimensions are in mm.

Figure 4 presents a schematic of the part that is 75 mm long, 12 mm tall, and 5 mm wide, with 7 mm tall 'legs' that form into 45° overhangs below a solid structure. The recoating direction starts at the pointed end with the 45° taper and proceeds to the left. A stereolithography (STL) file for the individual part can be downloaded from the AM-Bench challenge description website [4] to allow the part to be manufactured for subsequent studies.

Four parts were fabricated on a single substrate in a build. The build was repeated twice in each material to produce eight total parts of each material. The substrates used were 100 mm squares, 12.7 mm thick, mounted to the middle of the build area using four ¼in.-20 cap screws. The substrates were produced from nominally the same alloy (IN625 or stainless-steel 15-5) as the powder used in the build.

**Fig. 5:**
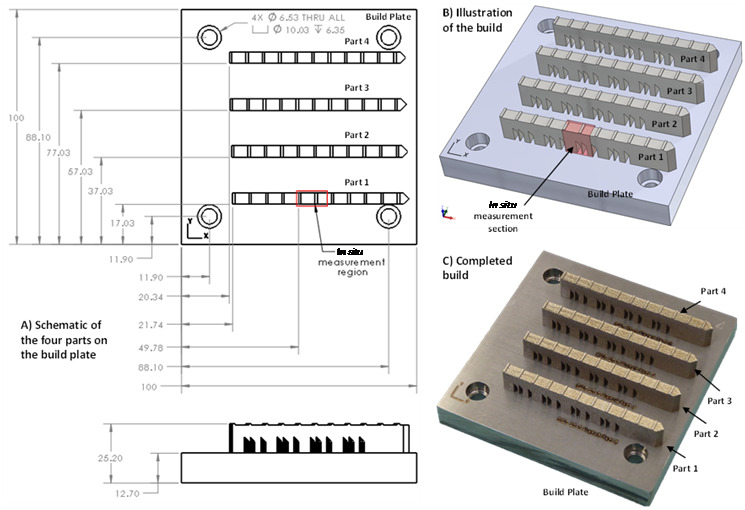
Build layout used for both materials.

Four parts were fabricated on each build plate, as shown in Fig. 5. Each part was identical. They were spaced by 20 mm along the *y*-axis, and they were offset from each other along the *x*-axis by 0.5 mm so that the recoater blade progressively engaged each part. The parts were fabricated in the order they were labeled (part 1 first, part 4 last). An STL file of the build plate and four parts can be downloaded from the AM-Bench challenge description website [4].

## 3.3 Metal Powder

All IN625 and 15-5 powders were obtained from the same respective lots and kept sealed in original shipment containers until use. Virgin powder was used in each build. Mill Test Certifications supplied by the manufacturer for the IN625 and 15-5 powders are available for download [4]. The measured particle size distribution (PSD) and chemical composition are provided in Table 1.

## Table 1 Particle size distribution and chemical composition of the metal powders used in the experiment.

**Table:** 

Attribute	IN625	15-5
Particle Size Distribution (PSD) - Samples were measured using a commercial dynamic image analysis instrument, average of three measurements. - Samples were taken from powder containers immediately after opening. - Samples were riffled before testing.	*D*_10_ = 16.4 µm *D*_50_ = 30.6 µm *D*_90_ = 47.5 µm	*D*_10_ = 20.4 µm *D*_50_ = 34.0 µm *D*_90_ =50.6 µm
Chemical Composition - Values in this table were taken from vendor-supplied data sheets, which utilized ASTM E1019[5] and ASTM E2823/#1479 [6]. - Compositions were also remeasured by a third party using ASTM E1019 - All composition measurements are in mass (weight) fractions.	C = 0.02% S = <0.005% N = 0.012% Mo = 8.82% Nb = 3.97% Co = 0.17% Fe = 0.81% Ti = 0.39% Mn = 0.04% Cr = 20.61% Si = 0.18% P = <0.10% Al = 0.3% Ni = 64.66%	Fe = 75.91% C = 0.02% Cr = 14.9% Cu = 3.9% Mn = 0.1% Mo < 0.1% N = 0.04% Nb = 0.3% Ni = 4.3% O = 0.03% P = < 0.01% S = < 0.01% Si = 0.5%

## 3.4 Scan Strategy

This section presents the scan strategy and scan parameters used in each build. The scan strategies and parameters for both IN625 and stainless-steel 15-5 were identical, including the laser power and speed settings. The only difference in the processing conditions was the recoater blade: A high-strength steel blade was used for the IN625 builds, whereas a ceramic blade was used for the stainless-steel 15-5 builds. For both materials, the recoater traveled at a speed of 80 mm/s.

**Fig. 6:**
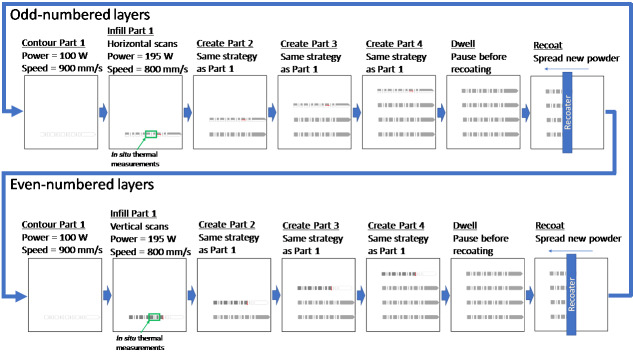
Illustration of the build strategy. This strategy was used to create both the IN625 and stainless-steel 15-5 parts.

Each layer consisted of a contour scan followed by an infill scan. Within each layer, the contour and infill of a part were completed before the next part began. During odd-numbered layers, the infill pattern consisted of horizontal scans (parallel to the *x*-axis) that were separated by 0.1 mm (hatch spacing). During even-numbered layers, the infill pattern consisted of vertical scans (parallel to the *y*-axis) that were also separated by 0.1 mm. During these infill scans, the beam offset was 0.03 mm, which means that the scan tracks began and ended 0.03 mm from the perimeter of the part. In between each layer, the build platform was lowered by 0.02 mm, so that a new layer of virgin powder could be spread across the powder bed. This process is outlined in Fig. 6 and summarized in Table 2. In addition, the odd and even layers are demonstrated in two different videos that can be downloaded at the AM-Bench website [4]. One video illustrates the scan strategy, while the other video is a recording made during creation of several layers from inside the build chamber.

## Table 2 Summary of the build parameters. The same parameters were used for each material.

**Table:** 

Variable	Value
Total number of layers	624
Layer height - Step change in build plate height between each layer	0.020 mm
Contour scan speed	900 mm/s
Contour laser power	100 W
Infill scan speed	800 mm/s
Infill laser power	195 W
Hatch spacing - Distance between adjacent scan vectors	0.100 mm
Laser spot size (diameter) - Laser spot was nominally Gaussian - Spot size was based on vendor-supplied values	0.10 mm
- Inert gas	Nitrogen
Oxygen level - Process would not begin with oxygen levels greater than 1.3% - A steady state of approximately 0.5% oxygen was typical for most layers	≈ 0.5%

## 3.4.1 Contour Scan Strategy

The contour of each feature on the part was scanned first using a programmed laser power of 100 W and a scan speed of 900 mm/s. For these parts, the beam offset was set to zero, which means that the center of the laser scan track aligned with the perimeter of the part. For example, the contour scan of the large legs (L1, L4, L7, L10), which were 5 mm squares, was performed with four 5 mm long scan tracks. The contour scan of the small legs (L2, L5, L8, L11) was created using two 5 mm long scans and two 0.5 mm long scans.

The number of contour scans and their timing depended on the features that were being created. For instance, as the 12 legs and the base of the part were being scanned in layers 1 through 250 (*Z* = 0.02 mm to *Z* = 5.00 mm), the laser-on times for legs of similar sizes were consistent. Laser timing was acquired by recording the laser-on/-off signal at a rate of 200 MHz using a Nicolet Odyssey XE oscilloscope for select layers and comparing the signal with the corresponding low-speed and high-speed videos of the process. However, since the order of the contouring operations and the starting location of each contour varied from layer to layer, the time between legs varied slightly (between 15.5 ms and 25.5 ms), depending on where the laser traveled to next to begin the subsequent contour scan. Furthermore, as the overhang structure began to form from layers 251 through 350 (*Z* = 5.02 mm to *Z* = 7.00 mm), the perimeter of the legs and base increased, necessitating a greater amount of time to fabricate these 13 features. Once the overhang features were complete, the individual leg sections merged, and only the bridge was fabricated in layers 351 through 600 (*Z* = 7.02 mm to *Z* = 12.00 mm); a single contour was required that took less time than the 13 individual contours. This information is shown in Table 3. Other than some variations between layers due to the contour scan sequence, there was no difference between the contour strategies for the even and odd layers.

## Table 3 Summary of the contouring scan timing for each feature of a part. The timing was the same for both materials.

**Table:** 

Features	L1, L4, L7, L10	L2, L5, L8, L11	L3, L6, L9, L12	Base	Bridge
Layers 1-250 Laser-on duration (ms)	22.4	12.4	16.8	50.3	N/A
Layers 251-350 Laser-on duration (ms)	22.4-26.67	12.4-16.67	16.8-21.11	50.3-54.7	N/A
Layers 351-600 Laser-on duration (ms)	N/A	N/A	N/A	N/A	112.5

## 3.4.2 Infill of the Odd-Numbered Layers

**Fig. 7:**
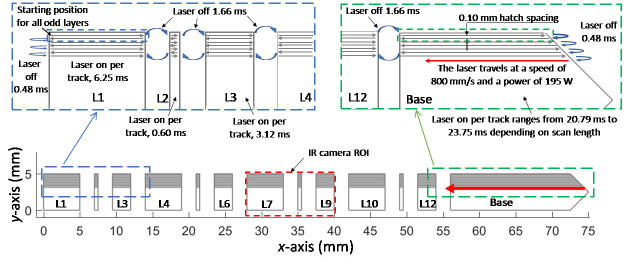
Description of the odd-numbered layer scan pattern and the laser-off time between each scan line. This was the same for both materials. Note that the number of scan tracks and the scale in each illustration are not accurate; this figure is intended to illustrate only the laser timing and spacing.

All odd-numbered layers were processed by the laser traveling at a programmed speed of 800 mm/s using a programmed power of 195 W. The laser scanned back-and-forth in the horizontal direction (parallel to the *x*-axis). The first infill scan line of each odd layer began at the upper-left corner of the part, in L1, and traveled to the right (+*x*), skipping from one feature to the next (with the laser shut off) along a constant *y* coordinate until it reached the end of the furthest feature to the right (the base), at which point the laser turned off, and the scan direction reversed. This process is illustrated in Fig. 7. Within each feature, the beam began and ended 0.03 mm from the left and right edges of the feature, respectively.

The laser-on time for each feature depended on the length of the scan line and the programmed laser travel speed (800 mm/s), as shown in Table 4. The table is divided into three sections. The first section describes the behavior when fabricating the first 5 mm of the legs, which had a constant cross section in each of these 250 layers. The second section provides information about the 350th layer, which was the final layer during the transition from the legs to the bridge using the 45° overhangs. The final section describes the timing of the bridge section, which spanned the entire width of the part. The laser-on time was calculated from measurements using an oscilloscope of the laser-on/-off command signal. These measurements were performed for layer 3 during a trial build and were assumed to be the same for all layers from 1 to 250.

## **Table 4.** Scan distance and laser timing for odd- numbered layers.

**Table:** 

Feature	Feature Length (mm)	Scan Line Length (mm)	Measured Laser-On Duration(mm)	Number of Scan Lines in Feature
Constant-cross-section legs (*Z* = 0.2 mm to *Z* = 5.0 mm)				
L1, L4, L7, L10 *(Layers 1–250)*	5.00	4.94	6.25±0.01	49
L2, L5, L8, L11 *(Layers 1–250)*	0.50	0.44	0.59 ± 0.01	49
L3, L6, L9, L12 *(Layers 1–250)*	2.50	2.44	3.12 ± 0.01	49
Base *Layers 1-250*	min: 16.50 *max:19.00*	min:16.49 *max:18.94*	min:20.75 ±0.01 *max:23.75 ±0.01*	49
Overhangs transitioning from the legs to the bridge (*Z* = 5.02 mm to *Z* = 7.0 mm)				
For layers 252-349, values were linearly interpolated between layer 250 and layer 350				
L1, L4, L7, L10 *(Layer 350)*	6.98	6.92	Not measured	49
L2, L5, L8, L11 *(Layer 350)*	2.48	2.42	Not measured	49
L3, L6, L9, L12 *(Layer 350)*	4.48	4.42	Not measured	49
Base *(Layer 350)*	min: 18.48 max: 20.98	min: 16.62 max: 18.94	Not measured	49
Bridge *(Layer 351-600)*	min: 72.50 max: 75.00	min: 72.49 max: 74.94	Not measured	49

The laser-off duration between scan tracks was also calculated from the oscilloscope measurements of the laser command signal. When transitioning between two features, such as L1 and L2 in Fig. 7, the laser was off for (1.66 ± 0.01) ms. This is consistent when transitioning between the different features during layers 1-205. In contrast, when the laser reached an end of the part, either L1 or the base, the off duration between the two adjacent scans was (0.48 ± 0.01) ms. Measurements were not acquired during the overhang layers, so no information is available regarding the duration the laser was off between features as the distance between them decreased because of the growing overhang. However, it is assumed that the laser-off duration at the ends of the part between two adjacent scan lines was the same: (0.48 ± 0.01) ms.

## 3.4.3 Infill of the Even-Numbered Layers

**Fig. 8:**
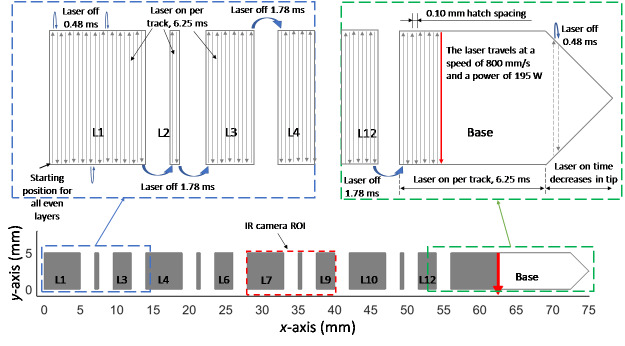
Description of the even-numbered layer scan pattern and the laser-off time between each scan line. This was the same for both materials. Note that the number of scan tracks in each feature is not accurate; this figure is intended to illustrate only the laser timing.

All even-numbered layers were processed by the laser traveling at a programmed speed of 800 mm/s and using a programmed power of 195 W. It scanned back-and-forth in the vertical (parallel to the *y*-axis) direction. The first infill scan line began at the lower-left corner of the part and scanned upward (+*y*). During these layers, the infill of each feature (*i.e*., legs) was completed before the laser began melting material in the next feature. The direction of each scan alternated regardless of whether the laser was continuing to scan a single feature or was transitioning between features. The scan lines began and ended 0.03 mm from the bottom and top edges of the feature. The laser-on times and laser-off times were consistent within features (excluding the right edge, which formed a point). However, the laser-off duration was longer between features. This information is presented in Fig. 8 and Table 5. Note that the number of scan lines in similarly sized features varied slightly.

## 3.4.4 Time between Parts within a Build

Within a layer, the time between the completion of the last infill scan line on a part and the beginning of the first contour of the next part ranged from 0.307 s to 0.363 s. This time variation was a function of the locations where the last infill scan concluded and where the first contour scan began. The contours were performed in a different order with a different starting position in each layer, thus causing the variation in time between parts.

## 3.4.5 Total Layer Time and Recoating

During the fabrication of the legs (*Z* = 0.02 mm to *Z* = 5.00 mm), the average layer time was 52 s. That is, 52 s passed from the time the first contour began on layer *n* to the time the first contour began on layer *n* + 1. Considering it took on average 26 s to scan the layer for all four parts, a significant amount of time was spent before the laser began melting material for the next layer. The longer-than-expected layer time of 52 s resulted from a dwell that was imposed at the end of each layer to allow the Additive Manufacturing Metrology Testbed (AMMT),^2^2
https://www.nist.gov/el/ammt-temps which has a longer duration recoating process, to replicate the build in future experiments.

## **Table 5.** Scan nominal distance and laser timing for even-numbered layers.

**Table:** 

Feature	Feature Width (mm)	Scan Line Length (mm)	Measured Laser-On Duration (ms)	Number of Scan Lines in Feature
Constant-cross-section legs (*Z* = 0.2 mm to *Z* = 5.0 mm)				
L1, L4, L7, L10 *(Layers 1-250)*	5.00	4.94	6.25 ± 0.01	L1, L10: 49 L4, L7: 50
L2, L5, L8, L11 *(Layers 1-250)*	5.00	4.94	6.25 ± 0.01	L2, L8, L11: 4 L5: 5
L3, L6, L9, L12 *(Layers 1-250)*	5.00	4.94	6.25 ± 0.01	L3, L9, L12: 24 L6: 25
Base *(Layers 1-250)*	min: 0.00 max: 5.00	min: 0.00 max: 4.94	min: 0.01 ± 0.01 max: 6.25 ± 0.01	49
Overhangs transitioning from the legs to the bridge (*Z* = 5.02 mm to *Z* = 7.0 mm)				
For layers 252-349, values were linearly interpolated between layer 250 and layer 350				
L1, L4, L7, L10 *(Layer 350)*	6.98	6.92	Not measured	49
L2, L5, L8, L11 *(Layer 350)*	2.48	2.42	Not measured	49
L3, L6, L9, L12 *(Layer 350)*	4.48	4.42	Not measured	49
Base *(Layer 350)*	min: 18.48 max: 20.98	min: 16.62 max: 18.94	Not measured	49
Bridge *(Layers 351-600)*	min: 72.50 max: 75.00	min: 72.49 max: 74.94	Not measured	49

Recoating was performed using a solid recoating blade. When processing 15-5 stainless steel, a ceramic recoating blade was used. In contrast, a high-strength steel (HSS) recoating blade was used when processing IN625. The different recoating blade materials are specified for the nickel alloy and stainless-steel alloy used in this study. An HSS blade cannot be used with the stainless-steel powder due to its tendency to magnetize and ultimately affect the quality of the spread powder layer. In both cases, the recoating blade spread powder across the powder bed surface at a speed of 80 mm/s.

## 4. Data Files

The data set consists of 122 compressed zip files and two MATLAB functions. Each compressed file contains example plotted thermal videos and MATLAB data structures, which contain the numeric measurement data for 10 layers. These zip files contain the example thermal videos and associated measurement data for all 624 layers of the part during two separate builds. The name of each compressed file briefly describes the study, the layers, and the features being manufactured in those layers. For example, consider the following zip file name:

"NIST_AMBench_625_Build1_Layers_171-180_LEGS.zip."

This file contains data related to the NIST AM-Bench study. The material is IN625, and the measurements were acquired during the first of two builds (using that material). The zip file contains the videos and MATLAB data structures for layers 171 through 180. During these layers, the legs are being constructed.

As stated earlier, two MATLAB functions are also provided. The first function is called "MakeRadiantTempThermalVideo.m" and was used to create the thermal videos from the MATLAB structures. It is included in the data set to allow the user to better understand how to use the data by stepping through the function. The second function is called "ConvertToTrueTemp.m" and allows the radiant temperature to be calculated based on an assumed emissivity correction factor. This will be detailed in Sec. 4.3.

If users do not have a MATLAB license, the MATLAB data structures (*.mat) and functions (*.m) may be opened using Octave (https://www.gnu.org/software/octave/), a free computational software based on the GNU General Public License.

## 4.1 Thermal Video Descriptions

The other files that are included in each zip file are video files in MP4 format that provide an example preview of the thermal imager data in formatted plots with associated file metadata displayed. These may be useful to users as a reference to check their own video or data processing. Considering the camera ROI, only legs 7, 8, and 9 are measurable (refer to Fig. 1, Fig. 4, and Fig. 5). Each video name corresponds to the test name. The full field of view of the camera is shown in the videos, but only radiant temperatures from 550 °C to 1050 °C are displayed. The camera is sensitive to slightly lower temperatures, but nonlinearity and noise become issues. The camera saturates at temperatures between 1050 °C and 1100 °C, depending on each pixel. However, for the sake of simplicity, the upper temperature is limited to 1050 °C. Figure 9 presents an example video frame.

## **Fig. 9.** Example frame from the thermal video. Each component is labeled (A through G) and described in the text.

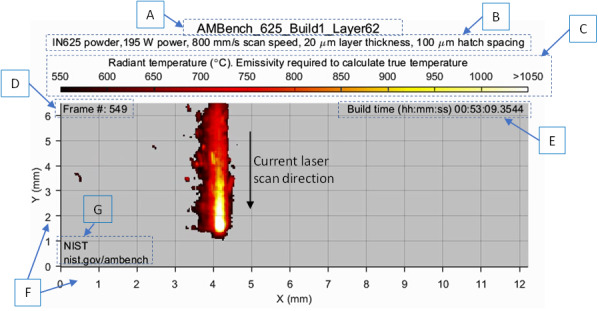



In this video, the laser is scanning down (*-y*) while creating leg 7. The previous scan track was beside it on the left, and the next scan track will be beside it on the right. Each video displays information about the layer and frame number. The header of the video contains the file name used to create the video (A). Similar to the zip file name example presented earlier, each video file name describes the test, material, build number, and layer number. This corresponds to the associated MATLAB data file. The header also contains information about the process (B). The process information describes the material, laser power and scan speed (excluding any contour scans), layer thickness, and hatch spacing.

Each video was created using the same fixed radiant temperature scale (C). Note that this is radiant temperature and not true temperature. The emissivity of the surface is required to calculate true temperature, as will be discussed in Sec. 4.3. The scale is limited to radiant temperatures between 550 °C and 1050 °C, since this is the practical range of the camera, as discussed earlier. Any radiant temperature equal to or greater than 1050 °C is displayed as white. Radiant temperatures below 550 °C are not displayed and are shown as a gray color, as evident in the thermal video frame.

Within the thermal video frame, the frame number (D) and build time (E) are displayed in the upper left and right, respectively. The frame number increases in increments with each new frame in the thermal video. For each layer, it begins at 1 and increases by 1 for each frame. This frame number correlates to the third dimension of the radiant temperature field in the MATLAB structure (refer to Sec. 4.2) to enable the user to analyze the data for a specific frame of interest. The build time refers to the time that has elapsed since the first instance the laser turned on in the first layer. Caution must be used when interpreting the thermal video, since the camera occasionally dropped frames (did not record some) or frames were excluded from the video file because there was no material in the region of interest above the detectable range of the camera. The frame number displayed in the video does not account for this, but the displayed build time does account for skipped frames.

The *x* and *y* axes (F) indicate the relative coordinates, in mm, within the region of interest of the video. These dimensions are not relative to the part or the machine coordinates. Please refer to the part schematic to determine the locations in the video that correspond to the local part coordinates. The dimensions of the *x* and *y* axes were calculated from the number of pixels in each axis (360 and 126, respectively) and the corresponding iFOV (which is provided in the MATLAB structure, which is discussed in Sec. 4.2).

Finally, in the lower-left corner of each video frame, the NIST logo and the AM-Bench website are displayed (G). This information is displayed so that the original source is known in case the videos are shared among various users.

## 4.2 MATLAB Data Structure Descriptions

This MATLAB data file contains a data structure for the layer of interest. If a MATLAB license is not available, the data structures may be opened using Octave (https://www.gnu.org/software/octave/), a free computational software based on the GNU General Public License.

Figure 10 provides an example of the structures contained in the data file and the variables within each data structure. These data structures can also be imported into other scientific programming languages, such as Python. When doing so, the field names within the structure may not transfer, and the fields will only be identified as numbered objects. However, the order should be the same as it is presented here. To verify which data point is associated with each imported object, import the file "AMBench_625_Build1_Layer62.mat" from the compressed file "NIST_AMBench_625_Build1_Layers_061-070_LEGS.zip" and compare the object data types, sizes, and values to the structure fields shown in Fig. 10. Each of these structure fields will be described in the following paragraphs. Please note that in the bottom three rows, the variables are too large for MATLAB to display in this window, and, consequently, the variable sizes and format are displayed in blue font.

## **Fig. 10.** Example of the structures in the MATLAB data file and the variables within each structure.

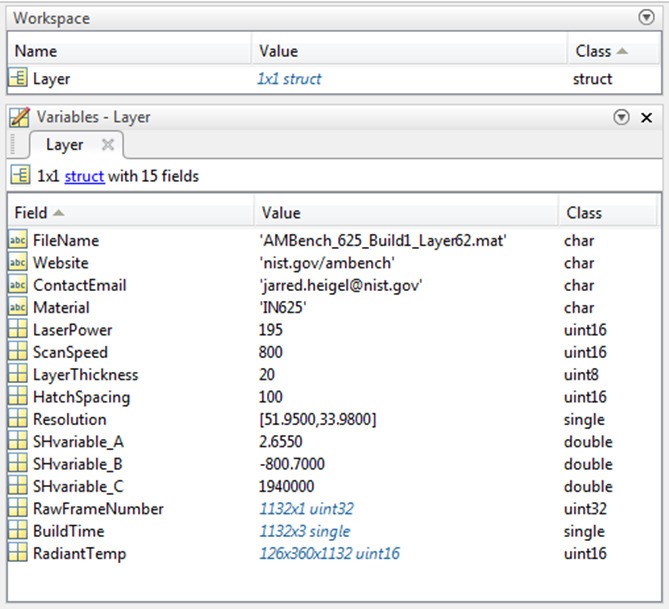



The first three fields are strings that provide information about the file and its source. The first field, called "FileName," is the filename of the MATLAB structure for the layer. It also relates to the video file name. The "Website" field is a string that informs the user of the website from which these data can be found and downloaded. Its inclusion ensures that even if the data file is shared between researchers, the original source is known. The third field, titled "ContactEmail," shares the email address of the NIST researcher responsible for the data.

The next five fields describe the process. "Material" is a string that gives a brief description of the type of powder used in the study. Greater detail on powder composition and size distribution can be found at the experiment description website [4]. "LaserPower" and "ScanSpeed" are integers that describe the programmed laser power and scan speed for the infill scans. These values are in the units of W and mm/s, respectively. "LayerThickness" is an integer that describes the distance, in µm, that the build platform moved down between layers. "HatchSpacing" is an integer describing the programmed distance, in µm, between adjacent laser scan tracks of the infill scan tracks.

"Resolution" is a single precision number that describes the iFOV of each pixel. The larger value describes the iFOV in the *y* direction (51.95 µm/pixel), while the smaller value describes the iFOV in the *x* direction (33.98 µm/pixel). The iFOV is not square because the camera observed the build plane at an angle of approximately 41°. These values were the same for each file (layer) within a build. However, between builds, the values may have changed slightly as a result of slight inconsistencies in the camera position when it was moved to allow access to the build chamber and repositioned in preparation for the new build.

The next three fields are double precision variables called "SHvariable_A," "SHvariable_B," and "SHvariable_C," and they provide the values of the *A*, *B*, and *C* variables from Eq. (1), respectively, which were obtained from the black-body calibration. These values can be used to convert from the radiant temperature provided in the data set to true temperature, as will be described in Sec. 4.3.

The final three fields in the structure provide measured radiant temperature and the timing information of each frame. The "RadiantTemp" field is a three-dimensional array containing the radiant temperature (*T*_radiant_), in °C, measured during the test. Since the emissivity of the solidified surface is unknown at this time, only the radiant temperature is provided. In the example shown in Fig. 10, the layer consists of 1132 frames, and each frame is 360 pixels wide by 126 pixels tall. This field only contains frames with a measurable temperature (any pixel in the ROI greater than or equal to 550 °C). Many frames were removed from the raw camera signal because the laser was off, or it was scanning outside of the ROI, and none of the material within the ROI was hot enough to be measured by the camera.

The fields "RawFrameNumber" and "BuildTime" must be used to understand when frames were skipped and to be able to relate the thermal data to time. "RawFrameNumber" is a one-dimensional array with a length equal to the number of frames in "RadiantTemp." Each value provides the frame number from the raw camera video of the layer from which the "RadiantTemp" frame was extracted. "BuildTime" is a two-dimensional array, with a length also equal to the number of frames in "RadiantTemp." Each row describes the time (hours, minutes, and seconds) from the first time the laser turned on during the first layer.

## 4.3 Description of the MATLAB Functions

There are two MATLAB functions provided. "MakeRadiantTempThermalVideo.m" will recreate the example MP4 thermal videos provided in the data set. The input of the function is the "Layer" data structure contained in each MATLAB data file. Stepping through the function should help to understand how the data in the structure were used. The second function is called "ConvertToTrueTemp.m" and can be used to convert the radiant temperature measurements that are provided in the "Layer" structure into true temperature.

The function "ConvertToTrueTemp.m" requires two inputs: the "Layer" MATLAB structure and an assumed emissivity correction factor. At this time, it is the responsibility of the user to assume an effective emissivity. In this function, the radiant temperature is first converted back to $S_{\text{meas}}$ using Eq. (2) and the values of *A*, *B*, and *C* provided in the "Layer" structure. Then, the true temperature is calculated using Eq. (1) and Eq. (3) and the assumed effective emissivity.

## 5 Impact

The purpose of this data set is for the validation of process models that simulate the fabrication of the AM-Bench 2018 bridge structure; however, care must be taken because emissivity of the surface during processing must be known to calculate the true temperature of each layer. Since the microstructure, strain, and distortion are a direct result of the thermal history the material experiences during the process, accurate models of those phenomena likely depend on accurate, validated, thermal process models. In addition to model validation, the measurements of the thermal history can be used by material scientists to understand the phenomena observed in the experiment microstructure.
